# A direct PCR approach with low-biomass insert opens new horizons for molecular sciences on cryptogam communities

**DOI:** 10.1128/aem.00024-24

**Published:** 2024-02-13

**Authors:** Patrick Jung, Laura Briegel-Williams, Lina Werner, Emily Jost, Matthias Schultz, Dennis J. Nürnberg, Martin Grube, Michael Lakatos

**Affiliations:** 1 Department of Integrative Biotechnology, University of Applied Sciences Kaiserslautern, Pirmasens, Germany; 2 Institute for Plant Science and Microbiology, Herbarium Hamburgense, University of Hamburg, Hamburg, Germany; 3 Institute of Experimental Physics, Freie Universität Berlin, Berlin, Germany; 4 Dahlem Centre for Plant Sciences, Freie Universität Berlin, Berlin, Germany; 5 Institute of Biology, University of Graz, Graz, Austria; Michigan State University, East Lansing, USA

**Keywords:** cyanobacteria, toxins, lichen symbionts, green algae, biocrusts

## Abstract

Molecular sequence data have transformed research on cryptogams (e.g., lichens, microalgae, fungi, and symbionts thereof) but methods are still strongly hampered by the small size and intermingled growth of the target organisms, poor cultivability and detrimental effects of their secondary metabolites. Here, we aim to showcase examples on which a modified direct PCR approach for diverse aspects of molecular work on environmental samples concerning biocrusts, biofilms, and cryptogams gives new options for the research community. Unlike traditional approaches, this methodology only requires biomass equivalent to colonies and fragments of 0.2 mm in diameter, which can be picked directly from the environmental sample, and includes a quick DNA lysis followed by a standardized PCR cycle that allows co-cycling of various organisms/target regions in the same run. We demonstrate that this modified method can (i) amplify the most widely used taxonomic gene regions and those used for applied and environmental sciences from single colonies and filaments of free-living cyanobacteria, bryophytes, fungi, and lichens, including their mycobionts, chlorobionts, and cyanobionts from both isolates and *in situ* material during co-cycling; (ii) act as a tool to confirm that the dominant lichen photobiont was isolated from the original sample; and (iii) optionally remove inhibitory secondary lichen substances. Our results represent examples which highlight the method’s potential for future applications covering mycology, phycology, biocrusts, and lichenology, in particular.

**IMPORTANCE:**

Cyanobacteria, green algae, lichens, and other cryptogams play crucial roles in complex microbial systems such as biological soil crusts of arid biomes or biofilms in caves. Molecular investigations on environmental samples or isolates of these microorganisms are often hampered by their dense aggregation, small size, or metabolism products which complicate DNA extraction and subsequent PCRs. Our work presents various examples of how a direct DNA extraction and PCR method relying on low biomass inserts can overcome these common problems and discusses additional applications of the workflow including adaptations.

## INTRODUCTION

Besides seed plants, cryptogams can be equally important to primary production in nearly every habitat. The term cryptogams comprises bryophytes, fungi, eukaryotic algae, prokaryotic cyanobacteria, and lichens as their symbiotic life forms. These diverse organismic groups not only commonly act as single players but also interact and contribute, in varying proportions, to diverse aspects. For example, biological soil crusts (biocrusts) (1), microbial biofilms (2), pathogens (3), toxic algal blooms (4), bioweathering agents (5), agriculture (6), or potential terraforming and space-related visions (7, 8).

For all the above-mentioned topics and sciences, DNA sequencing is a powerful tool for classifying the different components of cryptogam communities. The advent of sequencing technologies and the incorporation of sequence data have resolved long-standing challenges especially in the taxonomy of fungi, lichens, and microalgae (cyanobacteria and algae).

However, they recurrently face a fundamental problem: a big proportion of fungi, cyanobacteria, and algae are very difficult to isolate from *in situ* material (9). Many isolation techniques have been developed, but species-specific conditions must be initially determined (10, 11), and even if these approaches are successful, their exceptionally low growth rates can hamper DNA isolation and sequencing attempts. This also relates to some important groups of fungi such as members of the Dothideomycetes, where single colonies take, on average, half a year to reach a diameter of a few millimeters [e.g., see reference (12)], which is then still not sufficient to combine sequencing and isolation techniques.

All of the above addressed cryptogams are well known for their symbiotic interactions that often exceed our understanding of symbiosis and the single roles of the partners within this symbiosis. For example, lichens were recently redefined as a self‐sustaining microecosystem outlined by an exhabitant fungus including one or more extracellular photosynthetic partners and an indeterminate number of other microscopic organisms (13), which highlight their microbial complexity. This multi-partner symbiosis also includes so-called borderline lichens formed by the little understood *Lichenothelia* fungi that facultatively interact with surrounding green algae but do not form a lichen thallus (14). Other examples are lichens such as *Thermutis velutina*, species of *Lichinodium* or *Coenogonium linkii*, where single fungal hyphae are inseparably wrapped around a single cyanobacterial (15–17) or algal filament (18), respectively.

Besides the fungal part of the symbiosis, similar problems concern the photobiont of a lichen comprising either green algae (chlorobiont) or cyanobacteria (cyanobionts) or both (tripartite lichens). After decades of focused work, it has only recently been shown that both photobiont types comprise high degrees of diversity that were hidden due to a lack of existing isolates (19, 20).

Another layer of cryptogam complexity is added by biocrusts, which are composed of, in various proportions, mainly phototrophic microorganisms colonizing the upper few millimeters of soil in, e.g., arid regions (1). Here, interactions between the matrix provided by cyanobacterial extracellular polymeric substances (EPS) (cyanosphere), the cyanobacterium itself, and heterotrophic bacteria have been highlighted (21), but also lichens (22) or mosses (23) can be dominating aspects of biocrusts, including their symbiotic partners.

Comparably complex microbial associations dominated by photoautotrophs can often be found in cave environments where cyanobacteria, green algae, and heterotrophic microorganisms form biofilms that often display a three-dimensional organization (24). For such samples, isolation techniques often fail due to the unique combinations of abiotic conditions such as mineral-rich runoff water, low light, specific light qualities, or habitat-specific temperature amplitudes (25, 26).

Hence, there is a need for alternative culture-independent approaches for sequencing DNA extracted from environmental samples. While metabarcoding has become a routine approach to characterize the organisms in complex samples, it frequently bears multi-fold drawbacks: a fraction of sequences can neither be clearly assigned to a known group nor attributed to a microscopic component, and as the organisms are not isolated, it is hard to draw conclusions about their ecology. In addition, DNA extraction methods, in general, have specific efficiencies, depending on the types of (micro)organisms, which is still considered to be a huge bias during metabarcoding studies on species-rich and diverse samples (27, 28).

Irrespective of sequencing environmental samples or isolates, one common problem in DNA sequence-based studies on cryptogams is the difficulty in obtaining sufficient, high-quality DNA using standard extraction protocols with, e.g., cetyltrimethylammonium bromide (CTAB), or commercially available DNA extraction kits (29–31). The yields using these protocols are usually below 5 ng/µL, which can be explained by the nature of the cryptogams (32, 33). On the one hand, they excrete EPS made of C5 and C6 sugars, amino acids, alkali-insoluble/soluble hexoses, and chitinous amino sugars (32, 34) that weaken enzymatic lysis reactions, block sonication or columns, and thus limit the success of isolating total genomic DNA for downstream applications. On the other hand, lichens possess more than 1,000 known secondary metabolites that hinder not only DNA extractions but also PCR reactions (35, 36). Furthermore, commercially available DNA extraction kits usually take about 30 min up to several hours due to incubation steps in lysis buffers or other solutions. In addition, it is often difficult to obtain sufficient amounts of material for DNA extraction for many lichens in the first place. This is especially the case for crustose microlichens or most cyanolichens, e.g., of the order Lichinales. This issue is further intensified when the algal photobiont of a lichen sample should be isolated simultaneously to molecular work, and type material has to be submitted to a herbarium in order to track the history of the sample.

An ideal tool to overcome these aforementioned obstacles is an approach by which small amounts of biomass, such as a single-lichen apothecium containing the mycobiont only (37), or algal filaments or colonies [e.g., see reference (38)] are directly used for PCR or used for small-scale lysis prior to PCR. Several studies have developed and used such direct PCR approaches (37, 39, 40), but none of these has penetrated into the regular practice of molecular work on cryptogams. The absence of a commonly applied standard method for these specific applications often has to do with sophisticated workflows containing uncommon chemicals, specific instruments, or a methodology that only worked for a specific group or even genus of microorganism. Additionally, these methods are often cost effective but require special skills and are time consuming because they include various steps that are often inappropriate for large sample numbers.

In this article, we showcase the potential of a method that allows the isolation of DNA from 0.2- to 2.0-mm-sized pieces of biomass and the simultaneous co-amplification of phylogenetic and metabolic marker genes in a single standardized PCR program. This technique was modified, adapted, and applied to reveal the identity of the myco-, chloro- and cyanobionts of two lichen species and, additionally, to explore the diversity of biocrust samples, which include mosses, cyanobacteria, fungi, lichen mycobionts, and their green algal photobionts based on *in situ* material with the most widely used taxonomic primers. The approach was additionally used for previously isolated and characterized cyanobacteria where the partial 16S rRNA and full 16S–23S rRNA internal transcribed spacer (ITS) region was targeted. Furthermore, a broad spectrum of additional taxonomic markers for cyanobacteria, lichen mycobionts, and specific green algae were used, including primer sets for genes encoding for cyanotoxins and polyhydroxybutyrate (PHB) synthesis (bioplastic). We aim to introduce this method as a standard tool for various molecular genetic studies that include PCR and subsequent sequencing methods on cryptogams by demonstrating and discussing the outstanding potential for *in situ* studies. The presented methodology builds upon a commercially available kit, and as such, it is cost and time efficient and is suitable for large sample numbers, which makes this approach valuable for all manner of environmental samples and cryptogamic communities.

## MATERIALS AND METHODS

### Strains, culture conditions, and lichen material

All strains, lichen material, and material that were used for the different target co-cycling PCRs including the primers are indicated in Table S1. In detail, the tripartite lichen *Lobaria pulmonaria* (Germany) and the cyanolichen *Peltula* sp. (Namib Desert, South Africa) were chosen for the direct PCR approach.

Additionally, eight well-characterized cyanobacterial strains (*Synechococcus* sp. PCC 7009, *Oculatella crustae-formantes* PJ S28, *Hyella disjuncta* PCC6712, *Sociatus tenuis* SAG 26.92, *Chroococcidiopsis thermalis* PCC 7203, *Gloeocapsopsis diffluens* PJ S16, *Desmonostoc muscorum* PCC 7906, and *Symphyonema bifilamentata* DSM112338) were selected based on their fully available 16S rRNA gene region sequences.

Three cyanobacterial strains (*Gloeobacter* sp. Esc21.10, *Myxacorys chilensis* PJS 39, and *Nostoc* sp. LCyaPJ02) were isolated for this study following the workflow for free-living cyanobacteria as described in reference (41) and for the cyanobiont of the lichen *Lobaria pulmonaria* as described in reference (20).

In addition, the calcified sheath of the taxa *Geitleria* sp. Esc15.00 and biofilms formed by *Gloeobacter* sp. Esc21.10 were collected from the Escalon cave in Cantabria, Spain, which were also the source for the direct PCR approach as described below.

The direct PCR approach was also applied to the grit crust-biocrust type from the Atacama Desert (22) as well as to biocrust samples from Spitsbergen (42).

All isolates were kept in a culture room at 17°C, at a photosynthetic photon flux density of 30 µmol/m^2^/s at a light:dark interval of 16:8 hours for several weeks.

### Biomass preparation

The two lichens, *Lobaria parietina* and *Peltula* spp., were carefully washed for several minutes with sterile ddH_2_O, allowing the thalli to fully hydrate. *Lobaria pulmonaria* (~1 cm^2^) was dissected under a binocular stereoscope (Stemi 508; Zeiss, Oberkochen, Germany) using a sterile scalpel. For *Peltula* sp., several thallus squamules were picked under the stereoscope with sterile tweezers. The lichen fragments were transferred to sterile ddH_2_O and carefully cleaned under the stereoscope with tweezers in order to remove contaminating lichen fragments, mineral particles, and/or epiphytic algae. Three replicates composed of biomass fragments between 0.2 and 2.0 mm in size were picked from the lichen thalli of each lichen and directly transferred into the lysis buffer (Fig. 1.2A). In addition, three replicates from each lichen were prepared by transferring them into 100% acetone and incubating the fragments for 10 min (Fig. 1.2B) to remove possible secondary lichen metabolites such as usnic acid or parietin that can inhibit downstream processes. Such inhibitory substances are known from, for example, *Lobaria pulmonaria* (43). Acetone was chosen because it is known to extract various lichen substances while at the same time maintaining the lichen’s viability (44). Subsequently, the lichen fragments were transferred from acetone into sterile ddH_2_O and incubated for 10 min to remove the remaining solvent traces, which can be repeated as necessary. Finally, the lichen fragments were transferred to the lysis buffer.

**Fig 1:**
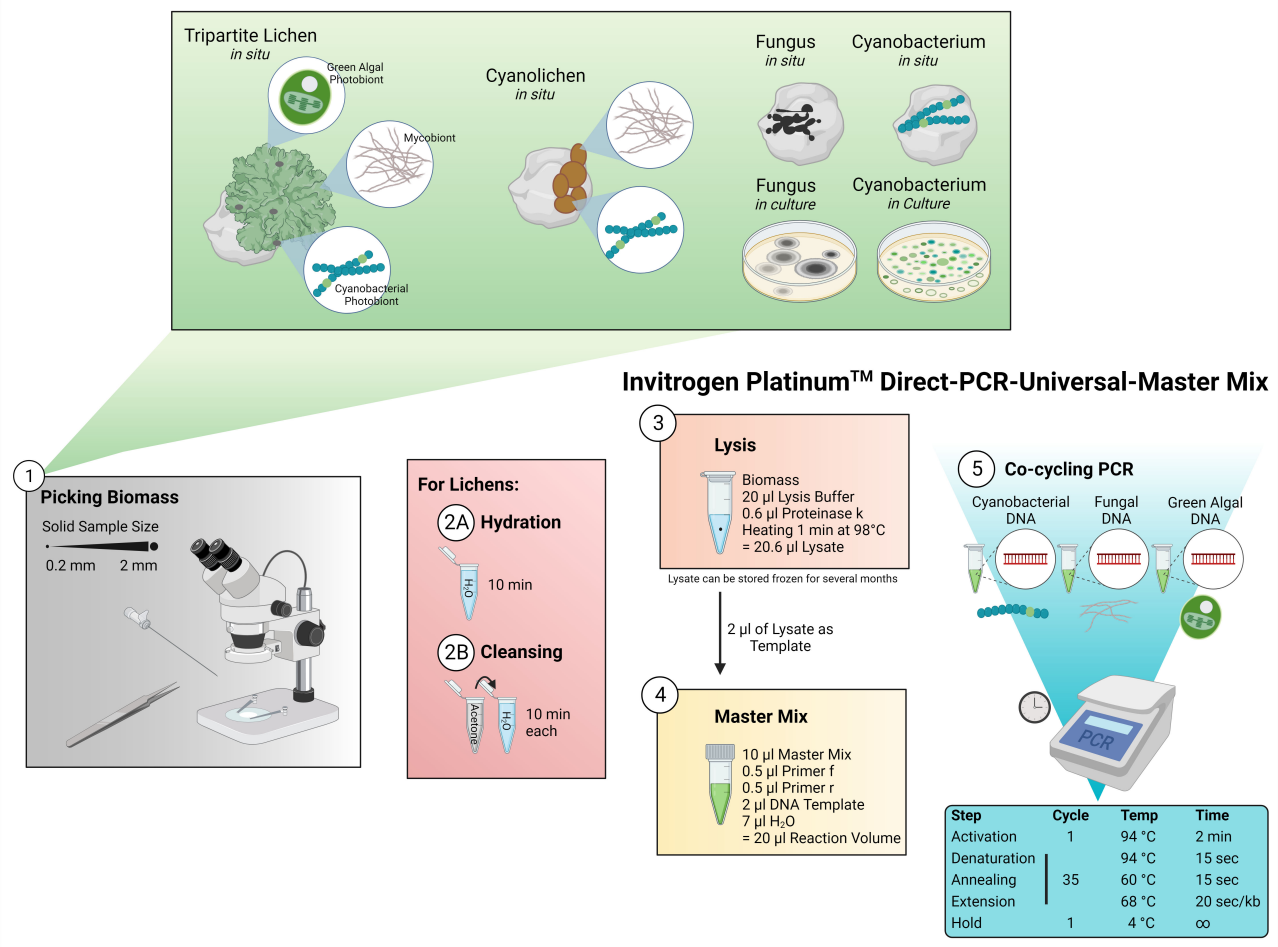
Applied workflow for the Platinum Direct PCR Universal Master Mix kit from Invitrogen. (1) Between 0.2- and 2.0-mm-sized pieces of biomass are picked from fungi (*in situ*, in culture) and cyanobacteria (*in situ*, in culture) under a binocular stereoscope using tweezers or needles which are then transferred into the lysis buffer. (2A) Biomass from lichens (*in situ*) is transferred into ddH_2_O for hydration prior to the lysis. (2B) Alternatively, the biomass from lichens can be incubated in acetone and afterward washed with ddH_2_O to remove secondary lichen substances that might inhibit downstream processes. (3) Lysate is prepared from 20-µL lysis buffer, 0.6-µL proteinase K, and the inserted biomass and is heated to 98°C for 1 min using a thermocycler. The lysate can be stored at −20°C for several months. (4) Master Mix (total volume of 20 µL) is prepared from 10-µL master mix, 0.5 µL of each primer, 2 µL of DNA template from the lysate and 7 µL ddH_2_O. 5 Co-cycling of several PCR targets is possible for fragments <2 kb using the standard PCR cycle following the instructions of the manufacturer. For longer fragments, the duration of the extension can be increased.

For cyanobacteria, a few filaments of *Geitleria* sp. were picked under a stereoscope with tweezers, while small proportions of *Gloeobacter* sp. were picked with a sterile needle directly from the biofilm material and placed in lysis buffer. The same procedure was used for cyanobacterial and fungal isolates which were taken directly from the agar plates.

The cyanobacteria, fungi, lichens, and bryophytes from biocrusts were picked with a sterile needle under the stereoscope and transferred into the lysis buffer.

### Cell lysis and PCR co-cycling

For the following steps, the Invitrogen Platinum Direct PCR Universal Master Mix (Thermo Fisher Scientific, Waltham, MA, USA) was used.

The lysate was prepared by mixing 20-µL lysis buffer, 0.6-µL proteinase K and the inserted biomass, followed by heating at 98°C for 1 min using a thermocycler (Fig. 1.3) as per method manual, although we recommend a 2-min lysis step for cyanobacterial lichens and eukaryotic green algae. The lysate can either directly be used for PCR or stored at −20°C for several months.

The PCR reaction mix was prepared from 10-µL Master Mix, 0.5 µL of each primer (10 pmol), 2 µL of DNA template (ca. 10–30 ng/µL) from the lysate, and 7-µL ddH_2_O for a total volume of 20 µL per reaction (Fig. 1.4) following the manual protocol. Lysates from the total lichens were used as DNA template for the mycobionts, cyanobionts, and chlorobionts.

Co-amplification of several loci was achieved by applying the standard PCR cycle suggested by the manufacturer (Fig. 1.5), addressing various target gene regions (Tables 1 and 2; Table S1). For fragments >2 kb, the duration of the extension was increased as recommended by the manufacturer, but this did not yield an amplification of the full 16S–23S rRNA ITS gene region of cyanobacteria. Instead, the original PCR cycle conditions for the selected primers (Tables 1 and 2) were found to be successful.

**TABLE 1:** Organism-specific PCR conditions used in this study

	Cyanobacteria		Fungi	Lichens			Bryophytes
				Mycobionts	Cyanobionts	Chlorobionts	
Marker	16S rRNA (V3-V4)	16S–23S ITS rRNA	ITS1	ITS1	16S rRNA (V3-V4)	18S–23S rRNA (partial)	Spacer trnM-trnV
Fragment length (bp)	390	2,300	1,300	1,300	390	1,400	750
Primer	CYA361f CYA785r	ptLSU C-D-rev SSU-4-forw	ITS1/ITS4 LR3/ITS5	ITS1 LR3	CYA361f CYA785r	Al1500af LR3	trnMF trnVR
PCR cycle	Standard	(45)	Standard	Standard	Standard	Standard	Standard

**TABLE 2:** Primer details

	Primer	Sequence	Reference
Cyanobacteria	CYA361f	GGA ATT TTC CGC AAT GGG	(46)
	CYA785r	GAC TAC WGG GGT ATC TAA TCC	
	ptLSU C-D-rev	GCC GGC TCA TTC TTC AAC	(45)
	SSU-4-forw	GAT CCT KGC TCA GGA TKA ACG CTG GC	
Fungi	ITS1	CTT GGT CAT TTA GAG GAA GTA A	(47)
	LR3 ITS4 ITS5	CCG TGT TTC AAG ACG G TCC TCC GCT TAT TGA TAT GC GGA AGT AAA AGT CGT AAC AAG G	(48)
Green algae	Al1500af	GCG CGC TAC ACT GAT GC	(49)
	LR3	CCG TGT TTC AAG ACG G	(50)
Bryophytes	trnVR	TYG AAC CGT AGA CAT TCT CGG	(51)
	trnMF	GCG ATA CTC TAA ACC ACT GAG	

Additional gene regions were successfully amplified, with the standard PCR or original cycles, and their products were sequenced (Table S2).

All obtained PCR products were checked by means of gel electrophoresis (E-Gel precast agarose gels with SYBR safe gel stain using the E-Gel Power Snap System of Invitrogen) and subsequently purified with the NucleoSpin Gel and PCR Clean-up Kit (Macherey-Nagel, Düren, Germany) following the DNA and PCR cleanup protocol. Purified PCR products were sent for Sanger sequencing by Azenta (GENEWIZ Germany, Leipzig, Germany) using the primers listed in Table 2, with the addition of the following primers for the 16S–23S rRNA ITS gene region of cyanobacteria: Wil 5, Wil 6, Wil 9, Wil 12, Wil 14, and Wil 16 (52).

### Bioinformatics

The generated sequences were assembled with Geneious Prime (v.2021.0.1) software package (Biomatters Limited, Auckland, New Zealand). Sequences of isolates which were created during this study (i.e., *Gloeobacter* sp., *Geitleria* sp., *Myxacorys chilensis*, *Lobaria pulmonaria*, *Peltula* sp., *Nostoc* sp., and *Chroococcidiopsis* sp.) and from the uncultured biocrust material were submitted to the National Center for Biotechnology Information (NCBI) GenBank, with accession numbers indicated in the corresponding phylogenetic trees or figure captions. For the lichen mycobionts, only the sequence from the hydrated DNA extraction was submitted to NCBI in order to avoid duplicates. The other sequences were not submitted to NCBI as they were found to be identical compared to the published sequences of the corresponding strains based on the NCBI GenBank BLAST tool; thus, a doubling of information was avoided.

The assembled and related sequences cited from GenBank were used for various phylogenetic analyses using the software Mega X (53). All alignments were prepared by applying the MUSCLE algorithm (54) and manually curated afterward. The evolutionary model that was best suited to each database was selected based on the lowest Akaike information criterion value and calculated in Mega X. In detail, the T93 + G evolutionary model was used for the alignment of the mycobiont and fungus phylogenies, RGT + G + I for the V3-V4 and the full 16S alignment of cyanobacteria as well as JC for the green algae phylogeny. The maximum likelihood method with 1,000 bootstrap replications was calculated with Mega X for each alignment and was visualized using iTOL (55).

## RESULTS

The described method showed a high success rate (>90% successful amplification checked by gel electrophoresis and subsequent high-quality Sanger sequencing) in amplifying various gene targets including the most widely used taxonomic primers and marker genes for cyanobacteria/cyanobionts, lichen fungi/mycobionts, and green algae/chlorobionts. An additional set of diverse primers for taxonomic marker regions and metabolite products was successfully used and is summarized in Table S2.

### Lichen symbionts taxonomic marker

**Fig 2:**
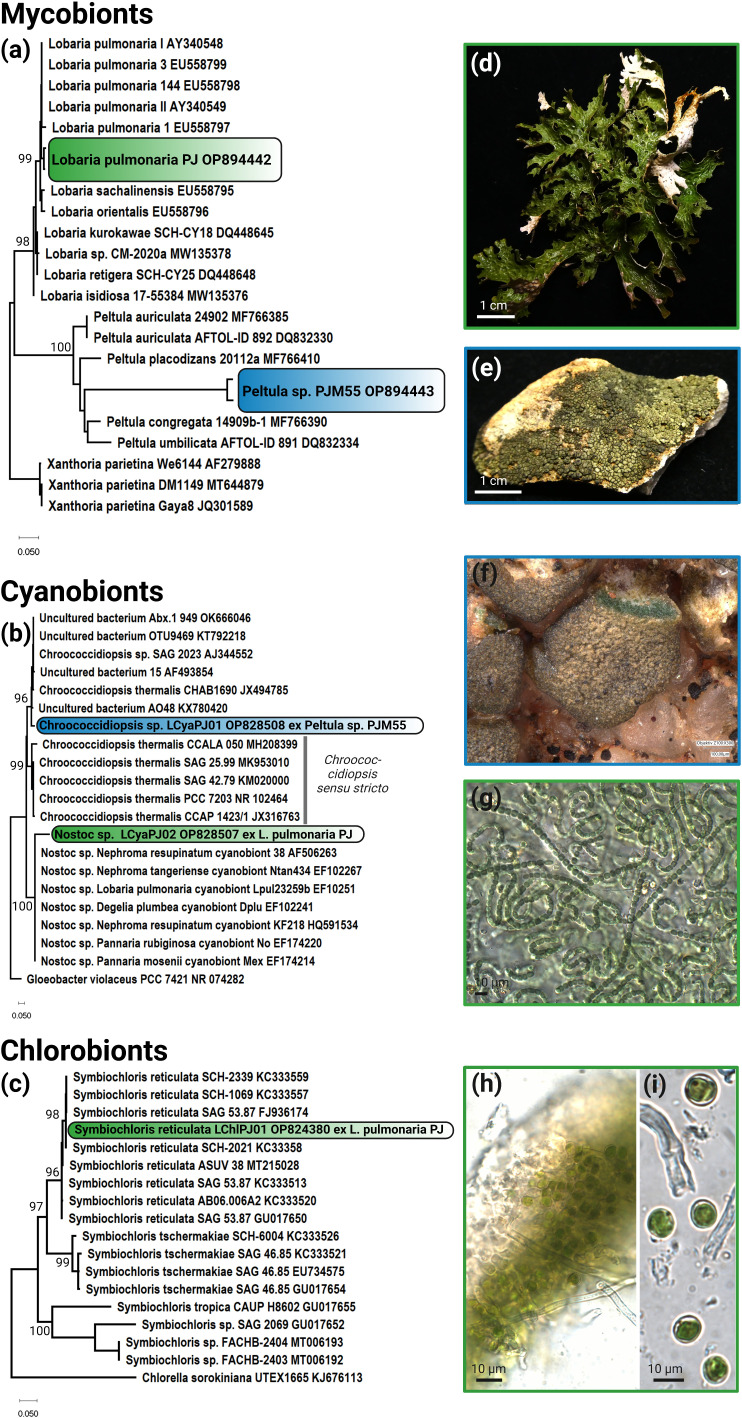
Phylogeny and micrographs of lichen symbionts derived from direct PCR. (a) The ML phylogenetic tree based on the nuITS gene region of the lichen mycobionts *Lobaria pulmonaria* and *Peltula* sp. and indicates the sequences that were generated by hydration of the lichen thallus fragments in ddH_2_O and those generated by an incubation step in acetone to remove secondary lichen substances. (b) The ML phylogenetic tree based on the V3-V4 gene region of the 16S rRNA gene of the cyanobionts *Nostoc* sp. and *Chroococcidiopsis* sp. derived from direct PCR of lichen fragments. Note that *Chroococcidiopsis* sp. falls outside of the *Chroococcidiopsis sensu stricto* clade [for comparison, see reference (20)]. (c) ML phylogenetic tree of the green algal photobiont *Symbiochloris reticulata* based on the partial 18S rDNA (SSU) to partial 26S (LSU) rDNA gene region derived from direct PCR of lichen fragments from *Lobaria pulmonaria*. (d) Photograph of *Lobaria pulmonaria*. (e) Photograph of *Peltula* sp. (f) Stereoscope close-up image of *Peltula* sp. with a cut squamule where the cyanobionts is visible. (g) microscopy of the isolated *Nostoc* sp. cyanobionts of *Lobaria pulmonaria*. (h) Microscopic cross section of *Lobaria pulmonaria* showing its chlorobiont *Symbiochloris reticulata* with cells of *Symbiochloris reticulata* freed from the lichen thallus in panel **i**.

In detail, small pieces of about 0.2–2.0 mm from the lichens were sufficient to extract DNA and amplify the marker genes for all three symbionts (Fig. 2). Only minimal differences (1- to 5-bp differences per >1 kb) were detected between mycobiont sequences generated from hydration in ddH_2_O or the pretreatment with acetone. Both sequences are shown in Fig. 2a, but only the sequence derived from the hydrated lichen material was submitted to NCBI GenBank. The nuITS gene sequence derived from *Lobaria pulmonaria* showed the highest similarity (99%) to other sequences from lichens of the same species. The nuITS sequence derived from *Peltula* sp. showed a lower similarity (>95%) but clustered with other sequences from *Peltula* representatives from the same habitat.

The V3-V4 region of the 16S rRNA from the cyanobionts and the mycobionts were amplified simultaneously. In addition, the same gene region was successfully amplified from the biomass of the isolated *Nostoc* sp. cyanobiont of *Lobaria pulmonaria* as proof for the correct isolation of the cyanobionts. Both sequences were identical, indicating that the dominant cyanobiont was isolated and belongs to the *Nostocales* (Fig. 2b). The generated *Nostoc* sequences were also identical (>99%) to those derived from the other gDNA extract of related lichens sharing *Nostoc* as photobionts. In the same way the cyanobionts of *Peltula* sp. showed the highest similarity to other *Chroococcidiopsis* sequences but turned out to group outside of the *Chroococcidiopsis sensu stricto* clade and instead formed a highly supported (99% and 96%) separate cluster (20).

The partial simultaneously derived 18S rDNA (SSU)–partial 26S (LSU) rDNA gene sequence of the green algal chlorobiont *Symbiochloris reticulata* of *Lobaria pulmonaria* was identical to other sequences derived from chlorobionts from the same lichen genus (Fig. 2c).

### Cyanobacterial taxonomic markers

**Fig 3:**
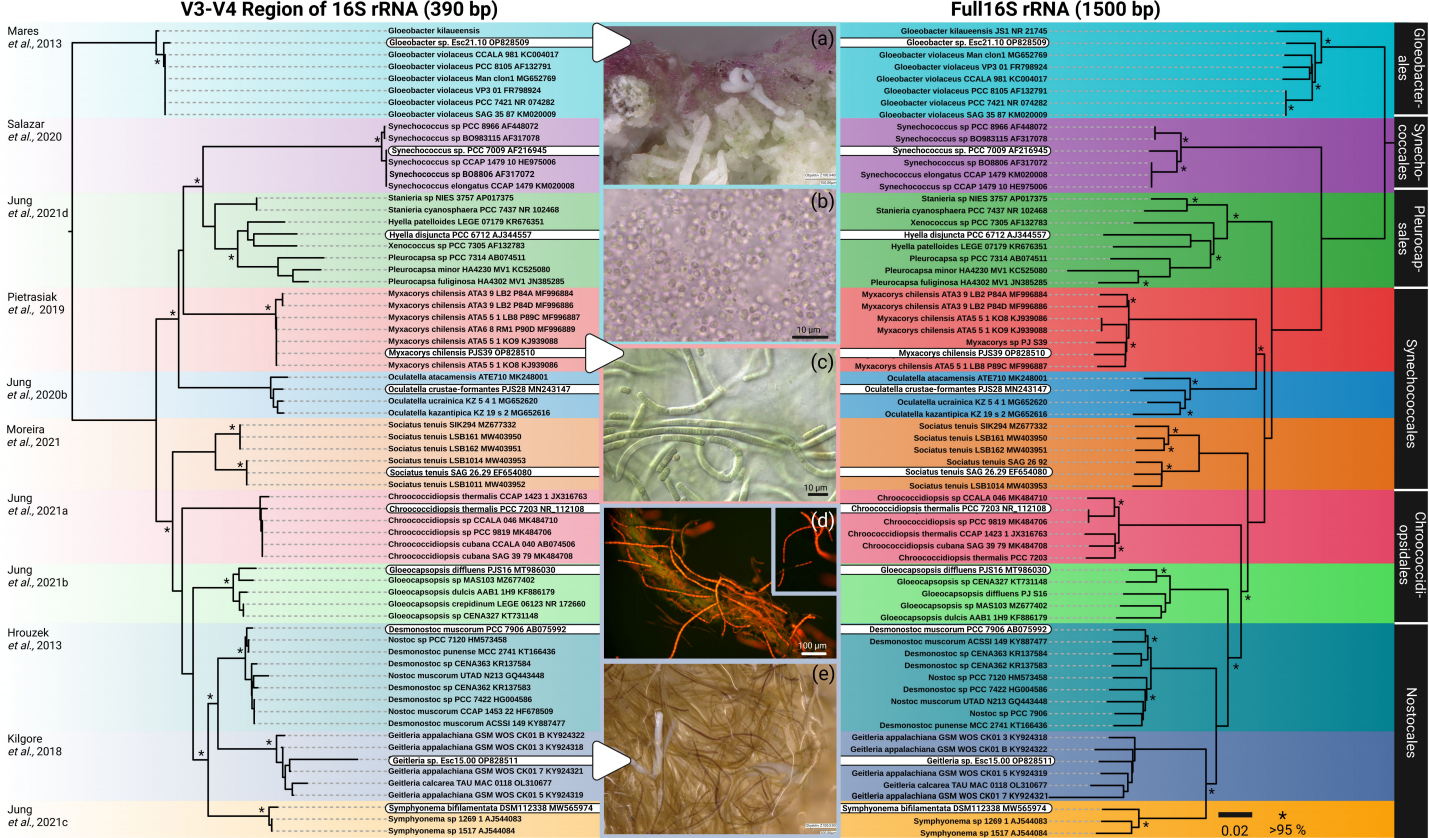
Phylogenetic tree of selected cyanobacterial strains reconstructed for the V3-V4 region of the 16S rRNA and the full 16S rRNA including micrographs. (Left) ML phylogenetic tree based on 390 bp covering the V3-V4 region of the 16S rRNA; (right) ML phylogenetic tree based on 1,500 bp covering the full 16S rRNA of the same strains. DNA sequences of *Geitleria* sp. and *Gloeobacter* sp. were generated from *in situ* material, while all other sequences come from culture isolates. The strains and DNA information of *Gloeobacter* sp. Esc15.00, *Geitleria* sp. Esc21.10 and *Myxacorys chilensis* PJS39 are novel. (a) Biofilms from which *Gloeobacter* sp. were *in situ* picked from for direct PCR and cultivation. (b) microscopic image of *Gloeobacter* sp. (c) Micrograph of *Myxacorys chilensis* PJS39. (d) Autofluorescence images of *Geitleria* sp. from *in situ* material intermingled with other cyanobacteria taxa showing the typical Y-branching. (e) calcified and non-calcified *Geitleria* sp. where single filaments were *in situ* picked from for direct PCR. See references (20, 26, 41, 56–63)for details.

In addition, the PCR of *in situ* material directly derived from cyanobacterial biofilms (*Geitleria* sp. and *Gloeobacter* sp.) was successful, resulting in high-quality sequence reads covering the V3-V4 region of the 16S rRNA and the full 16S rRNA gene region (Fig. 3). The recovered V3-V4 sequences of all cyanobacterial strains were found to be identical compared to corresponding regions within the generated full 16S rRNA gene sequences. During this study, the 16S rRNA gene sequences of the isolated strains *Geitleria* sp., *Gloeobacter* sp., and *Myxacorys chilensis* was generated and clustered with high support with other full 16S rRNA sequences from representatives of the respective genera.

### Biocrust taxonomic markers

**Fig 4:**
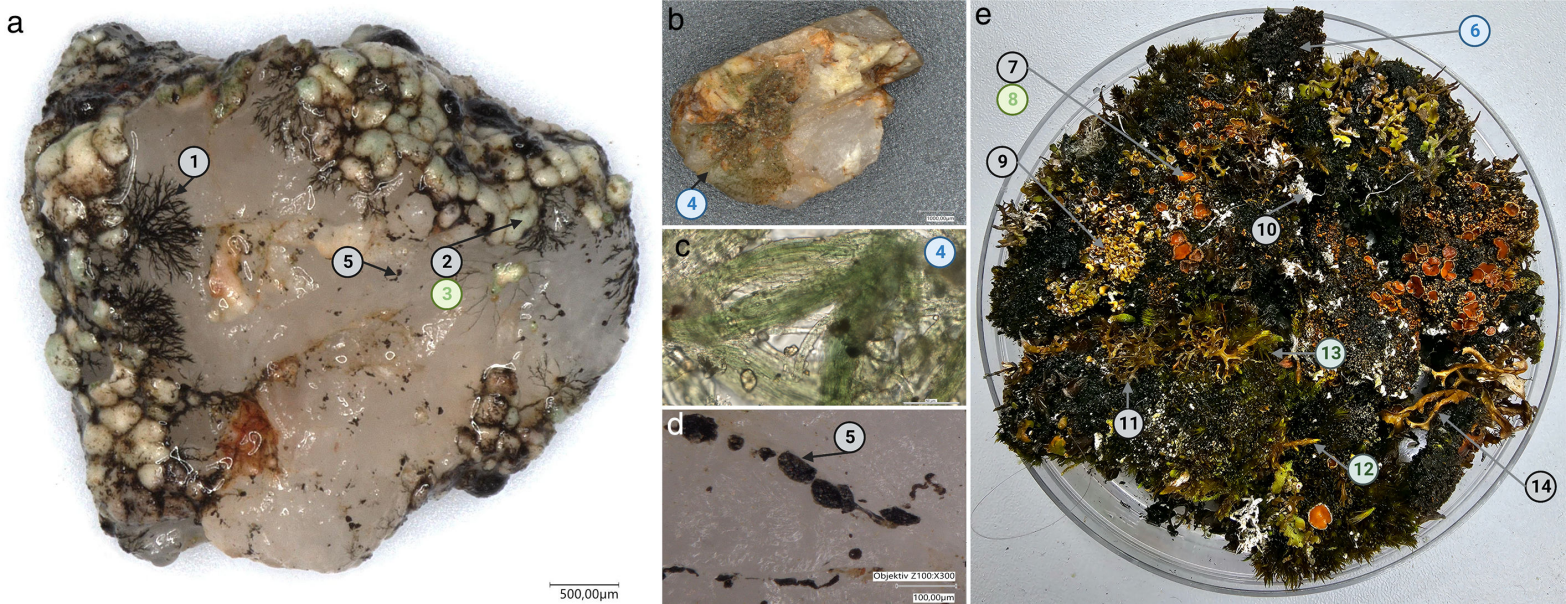
Direct PCR approach applied to biocrust samples. (a**–d**) grit crust from the Atacama Desert, Chile. (a) single grit crust stone from which the following DNA sequences were generated: 1, prothallus, *Buellia* sp. LC1-1; 2, lichen thallus *Buellia* sp. LC1-2; 3, photobiont from same sample, *Trebouxia* sp. LC1-3. (b) Grit crust stone with filamentous cyanobacteria from which sequence 4, *Microcoleus steenstrupii* KC2-4, was generated. (c) Microscopy image showing *Microcoleus steenstrupii* from 4. (d) Close-up of grit crust stone from panel A, from where sequence 5, *Constantinomyces* sp. MAGPIg26 OP894441, was generated. (e) Biocrust sample from the Arctic Spitsbergen in a 12-cm diameter petri dish from which the following sequences were generated: 6, *Nostoc* sp. D8C1-6; 7, *Psoroma hypnorum* F4-7; 8, photobiont of *Psoroma hypnorum*, *Trebouxia* sp. M17-8; 9, *Cladonia* sp. G4-9; 10, *Ochrolechia tartarea* D6-10; 11, *Cetrariella delisei* H4-11; 12, *Hypnum bambergeri*; 13, *Dicranum spadiceum*; and 14, *Cetraria islandica* FI5-14.

The presented method also generated high-quality DNA sequences from small grits (ca. 6 mm in diameter) from the Atacama Desert colonized by microscopic chlorolichens, free-living fungi, and cyanobacteria, as well as from larger biocrust samples from the Arctic made up of a highly diverse set of free-living cyanobacteria, bryophytes, and chlorolichens (Fig. 4). Due to the low levels of biomass required, individual biocrust organisms could be selected and picked for downstream analysis while avoiding direct contamination. Using other methods previously utilized by the authors for investigating cryptogams and biocrust communities, such as CTAB followed by phenol-chloroform-isoamyl alcohol purification (64, 65), this would not be possible due to the amount of biomass required for the recovery of suitable amounts of DNA.

## DISCUSSION

### Taxonomic notes

Cyanolichens encompass only about 10% of all known lichens (66) and often represent a neglected part of lichenology. However, the epiphyte *Lobaria pulmonaria* is frequently used as a model species to study, e.g., the factors affecting the population biology of lichens (67) and proteomics of the holobiont and its diverse microbiota (68). Our direct PCR approach can therefore be considered as a useful tool supporting scientific work on this model lichen (Fig. 2a). While *Lobaria pulmonaria* as a tripartite lichen with *Symbiochloris reticulata* as chlorobiont (Fig. 2c) and *Nostoc* spp. as nitrogen-fixing cyanobionts (Fig. 2b) received a lot of attention over the past decades, other cyanolichens such as members of the Lichinales are understudied. For these lichens, only few DNA sequences are available. Recently, the taxonomy of the cosmopolitan genus *Peltula* has been revised, which is always the case when newly generated DNA sequences become available, and shows that work on this genus is difficult (69). The lack of biodiversity information on these groups is reflected not only by the lichen mycobionts but also by their cyanobionts. For example, it was thought for decades that the unicellular cyanobacterium *Chroococcidiopsis* is the main cyanobiont of many Lichinales, but recent work has shown otherwise. Jung et al. (20), focused on the unicellular cyanobacterial symbiont taxonomy and demonstrated that no known isolated unicellular cyanobiont from Lichinales fell within the cluster of *Chroococcidiopsis sensu stricto* but rather formed yet undefined out-groups. This is again supported by this study, where the unicellular cyanobiont of a *Peltula* species whose V3-V4 gene region that was generated by the direct PCR approach based on lichen fragments clustered in the outgroup and not within *Chroococcidiopsis sensu stricto* (Fig. 2B).

In general, the V3-V4 region of the 16S rRNA has been proven to be an adequate marker gene for cyanobacterial phylogenies [e.g., see reference (70)]. Ample times it has indicated that cyanobacterial strains could be novel species, which was later shown to be the case after sequencing the full 16 S-23S rRNA ITS [e.g., see reference (41)]. Other advantages are that sequencing with one of the two primers is sufficient to cover the 390-bp fragment, which makes the new methodology cost and time efficient. However, it is more complicated when it comes to generating the full 16S–23S rRNA ITS gene region, which is required for the certain phylogenetic placement of cyanobacteria. Various primer pairs are available to cover this fragment, but the requirements for the quality of the DNA template are high as most of these PCRs amplify long fragments. In addition, the ITS gene region is a site of mismatches and high variability not only between taxa but also within one species due to copies of this region, which can contain different base pairs, resulting in mixed PCR products. Our applied workflow resulted in high-quality PCR products that could overcome these issues (Fig. 3). It allowed us to obtain sequences not only from the biomass of unialgal isolates but also from *in situ* material such as biofilms containing *Gloeobacter* or calcified sheaths with *Geitleria* as the dominant species. Obtaining *Geitleria* sequences is especially important because all prior isolation attempts of this cyanobacterium failed, as also previously reported for *Geitleria appalachiana* (26).

### Methodological notes

Since 2015, cyanobacterial taxonomy has been based on a polyphasic approach (71) which led, and still leads, to an impressive body of literature describing new species and untangling the complexity of this phylum. Our method will be a powerful tool for overcoming the many obstacles occurring during molecular work on these difficult-to-lyse bacteria. This spans from *in situ* material to isolates and also includes symbionts. Little is known about the phylogeny of lichen associating cyanobacteria because often only the protein coding *rbcL* gene region works for direct PCR approaches from lichens. This gene region leads to the formation of clusters in phylogenies but is inadequate for taxonomical purposes for various reasons (72). The presented method finally overcomes this issue and allows the amplification of the taxonomically important V3-V4 gene region and counter-checks that the main/dominant photobiont was isolated instead of epiphytic cyanobacteria.

In addition, the protocol has also been applied to a broad range of cryptogamic organisms and metabolic products including the cyanotoxins microcystin and saxitoxin B, cyanobacterial PHB, *Trentepohlia*-specific taxonomic markers, and protein-coding gene regions frequently used in lichen taxonomy (Table S2). These versatile applications demonstrate that the presented workflow can be useful for general molecular work on a diverse set of cryptogams, all of which currently represent great burdens with specialized methodologies that can now be simplified.

**Fig 5:**
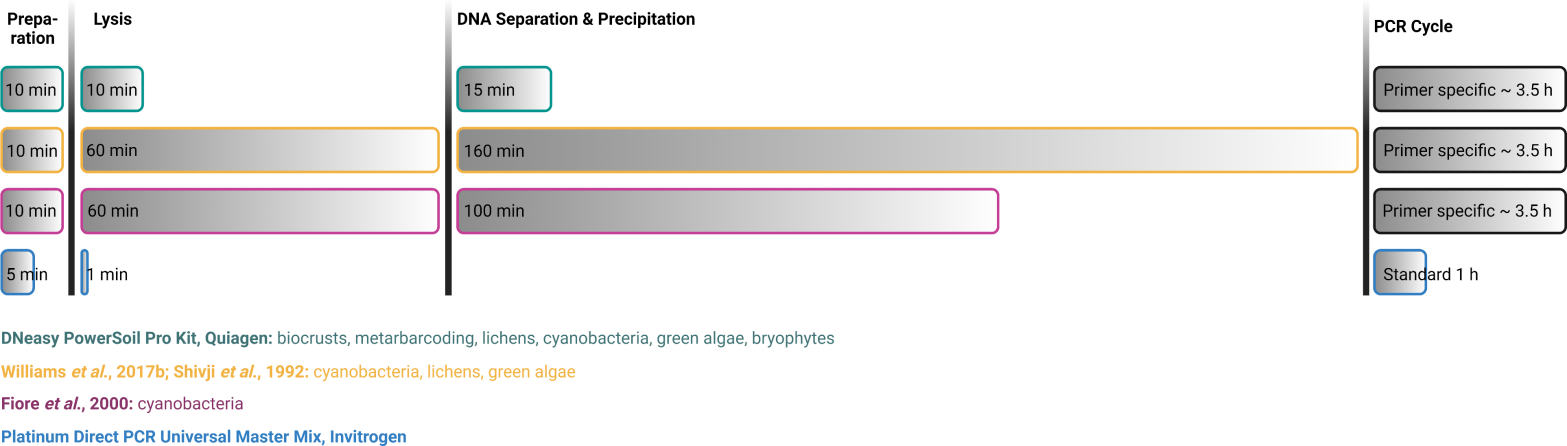
Comparison of DNA extraction methods and kits frequently used for cryptogams. See references (42, 73, 74) for details.

The method itself is advantageous in various ways: it is time efficient, for example, as the preparation and lysis of the sample take less than 10 min and the overall PCR cycle time using the standard cycle takes only 1 hour (Fig. 5). Other commercially available DNA extraction kits or methods state a duration exceeding often several hours because they rely on intensive incubation times during lysis (Fig. 5). Additionally, the original PCR cycles of most of the primers used in this study (Tables S1 and S2) run for 3.5 hours on average and thus block thermocyclers for a considerable time of the working day, while the presented method usually allows co-cycling of different primers and targets in 1 hour.

However, one of the biggest advantages of the method is the small amount of biomass, which enables successful molecular applications based on minute amounts of material that can be picked with tweezers under a binocular stereoscope. This alone gives a new outlining for various aspects of work on cryptogams, such as untangling complex samples where epiphytic or intra-thalline algae of lichens can be studied in order to uncover their role in the symbioses. The need for such small-scale techniques is, e.g., reflected in structurally challenging lichens such as *Lichina pygmaea* or borderline lichens such as *Collemopsidium*, where it is still difficult to pin down the Pleurocapsalean photobiont identity due to inadequate methods (75–77). As outlined previously, the CTAB method is still a standard technique for DNA extraction in lichenology, and molecular work on such small lichens is strongly hampered by the comparably large amounts of biomass. This also holds true for most DNA extraction kits, and as a result, elaborate cleaning steps involving Tween 20, Tween 80, or hydrogen peroxide, as in the protocol developed by reference (78), need to be applied prior to DNA extraction. This is usually done for a high number of samples because the success rate of DNA extraction and subsequent PCR reactions is low due to ongoing contamination or chemical reactions during cleaning (79).

### Outlook for cyanobacteria

Being able to use the standardized protocol for the amplification of multiple DNA fragments will furthermore allow screening for certain metabolic properties in parallel to the taxonomic approach. This includes genes for the synthesis of cyanotoxins (80) but also genes for the fixation of nitrogen (*nifH*) (81) and light acclimation responses (*apcE2*) (82), which may be especially important for cyanobacteria living in symbiosis and in association with cryptogams. While the impact of these organisms on the input of fixed nitrogen in pristine, high-latitude ecosystems has been shown (83), little is known about the importance in other habitats, and the effect of light variation, in particular, shading by the mycobiont and/or algal photobiont on the photosynthetic properties of the cyanobacteria, remains largely unknown. The presented method opens opportunities for resolving these questions.

### Outlook for fungi and (lichen) symbionts

The new method has potential to stimulate research especially in hitherto largely neglected groups of usually very small sized lichens (microlichens). This includes cyanolichens such as Lichinomycetes, but also other species-rich groups of lichens including Verrucariaceae (Eurotiomycetes) or Arthoniomycetes, as well notoriously difficult groups within the Lecanoromycetes dominated by microlichens. The technique may also be useful to study small lichenicolous fungi infecting the above-mentioned lichens. Furthermore, it is useful for studying questions of lichen individuality where boundaries between thalli are difficult to assess. Improved and accelerated description of novel species involving easy amplification of standard genetic markers as part of integrative taxonomic research will help to further consolidate existing classifications of lichen-forming fungi. This, in turn, will be influential for integrating lichens and their photobionts into ecological studies designed to better understand composition, interaction, function, usage, and future development of terrestrial ecosystems.

### Outlook for biocrusts and microbial biofilms

Low amounts of biomass inserts from *in situ* material were also successfully used for biocrust samples from the Atacama Desert of Chile as well as from the Arctic Spitsbergen (Fig. 4). Such samples are exceptionally challenging due to a high degree of diversity on a small scale and are thus often explored only by metabarcoding [e.g., see reference (84)] or as large-scale works presenting species lists for entire regions [e.g., see reference (85)]. The new method provides detailed information about single (micro)organisms that are part of biocrusts or biofilms based on very small fragments one can easily pick with a needle or tweezers, allowing the diversity of an intact biocrust to be explored. To our knowledge, this is the first time that multiple cryptogamic groups have been identified and sequences generated from small samples of biocrust material as the majority of researchers focus, and have expertise, on a certain organism group. Future studies could, for example, be carried out on the great diversity of microscopic lichens from biocrusts or saxicolous lichen communities from which genetic information on the mycobionts and their photobionts can be created. The point intercept method (86), which is commonly used when characterizing biocrusts, could also be supplemented with this methodology, allowing a far greater picture of biocrust diversity than is currently the norm from such studies. Along with the power of detailed photographs, an overview of the biodiversity will be possible with phylogenetics-based identifications assigned to exact specimens. This might appear more laborious than metabarcoding but comes with the great advantage that the generated genetic information can be consolidated, instead of a plethora of genetic information that cannot be assigned to the actual organisms from the samples.

### Limitations of the method

Although the presented method has been successfully applied for the first time by the author team for a large set of lichens including both their mycobionts and photobionts (87), we identified several limitations of the method regarding cryptogams. The manufacturer, for example, suggests an extension of the PCR cycle duration for target regions >2,000 bp, but we found some primer/organism combinations where neither the standard cycle nor the extension of the cycle time resulted in successful amplifications. Instead, original PCR cycles proposed by the authors of individual primer pairs or slight modification thereof were successful (Tables S1 and S2). In addition, some lichen groups such as certain red *Usnea* species from the Atacama Desert, which we also tested, showed to be incompatible with the kit irrespective of any pretreatment steps or primer adaptations, which may be due to red pigments of the lichens. Those examples indicate that the kit needs to be tested and individually evaluated whenever new organisms or primers are used.

## Data Availability

All generated DNA sequences were submitted to National Center for Biotechnology Information GenBank and can be found under the accession numbers indicated in the phylogenetic trees.

## SUPPLEMENTAL MATERIAL

Supplementary material aem.00024-24-s0001.docx Organisms and applied PCR conditions used in this study and successful additional primers and PCR conditions.

ASM does not own the copyrights to Supplemental Material that may be linked to, or accessed through, an article. The authors have granted ASM a non-exclusive, world-wide license to publish the Supplemental Material files. Please contact the corresponding author directly for reuse.
