# Supplementary material for: A direct PCR approach with low-biomass insert opens new horizons for molecular sciences on cryptogam communities
Source: Appl Environ Microbiol. 2024 Feb 13;90(3):e00024-24. doi: 10.1128/aem.00024-24 (PMC10952543; doi:10.1128/aem.00024-24)
Supplement: Supplementary material — Organisms and applied PCR conditions used in this study and successful additional primers and PCR conditions. [file aem.00024-24-s0001.docx]

**Supporting Information**

Article title:

Authors:

The following Supporting Information is available for this article:

**Table S1**

| **Type** | **Sample Name** | **Source** | **Origin** | **Target** | **Primers** | **Fragment Length [bp]** | **PCR Cycle** |
| --- | --- | --- | --- | --- | --- | --- | --- |
| Cyanobacterium | *Geitleria* sp. Esc15.00 | Calcified Filaments from Biofilm | Cave, Spain | Cyanobacterium | CYA361f CYA785r | 390 | Standard |
|  |  |  |  |  | ptLSU C-D-rev  SSU-4-forw | 2.300 | (71) |
| Cyanobacterium | *Gloeobacter* sp. Esc21.10 | Biofilm | Cave, Spain | Cyanobacterium | CYA361f CYA785r | 390 | Standard |
|  |  | Isolate |  |  | ptLSU C-D-rev  SSU-4-forw | 2.300 | (71) |
| Cyanobacterium | *Synechococcus* sp. PCC 7009 | Isolate | Pond, England | Cyanobacterium | CYA361f CYA785r | 390 | Standard |
|  |  |  |  |  | ptLSU C-D-rev  SSU-4-forw | 2.300 | (71) |
| Cyanobacterium | *Oculatella crustae-formantes* PJ S28 | Isolate | Biocrust, Arctic | Cyanobacterium | CYA361f CYA785r | 390 | Standard |
|  |  |  |  |  | ptLSU C-D-rev  SSU-4-forw | 2.300 | (71) |
| Cyanobacterium | *Myxacorys chilensis* PJ S39 | Isolate | Biocrust, Atacama Desert, Chile | Cyanobacterium | CYA361f CYA785r | 390 | Standard |
|  |  |  |  |  | ptLSU C-D-rev  SSU-4-forw | 2.300 | (71) |
| Cyanobacterium | *Hyella disjuncta* PCC6712 | Isolate | Freshwater, USA | Cyanobacterium | CYA361f CYA785r | 390 | Standard |
|  |  |  |  |  | ptLSU C-D-rev  SSU-4-forw | 2.300 | (71) |
| Cyanobacterium | *Sociatus tenuis* SAG 26.92 | Isolate | Biocrust, Negev Desert, Israel | Cyanobacterium | CYA361f CYA785r | 390 | Standard |
|  |  |  |  |  | ptLSU C-D-rev  SSU-4-forw | 2.300 | (71) |
| Cyanobacterium | *Chroococcidiopsis thermalis* PCC 7203 | Isolate | Soil, Germany | Cyanobacterium | CYA361f CYA785r | 390 | Standard |
|  |  |  |  |  | ptLSU C-D-rev  SSU-4-forw | 2.300 | (71) |
| Cyanobacterium | *Gloeocapsopsis diffluens* PJ S16 | Isolate | Hypolithic Biofilm, Atacama Desert, Chile | Cyanobacterium | CYA361f CYA785r | 390 | Standard |
|  |  |  |  |  | ptLSU C-D-rev  SSU-4-forw | 2.300 | (71) |
| Cyanobacterium | *Desmonostoc muscorum* PCC 7906 | Isolate | Freshwater | Cyanobacterium | CYA361f CYA785r | 390 | Standard |
|  |  |  |  |  | ptLSU C-D-rev  SSU-4-forw | 2.300 | (71) |
| Cyanobacterium | *Symphyonema bifilamentata* DSM112338 | Isolate | Biofilm on Stone, Switzerland | Cyanobacterium | CYA361f CYA785r | 390 | Standard |
|  |  |  |  |  | ptLSU C-D-rev  SSU-4-forw | 2.300 | (71) |
| Dothideomycete | *Constantinomyces* sp. MAGPIg26 | Stone | Stone, Atacama Desert, Chile | Fungus | ITS1  LR3 | 1.300 | Standard |
|  |  | Isolate |  |  | ITS1  LR3 |  |  |
| Tripartite Lichen | *Lobaria pulmonaria* | Lichen Thallus | Tree Bark, Lanzarote, Spain | Mycobiont (Fungus) | ITS1  LR3 | 1.300 | Standard |
|  |  | Lichen Thallus |  | Cyanobiont (Cyanobacterium) | CYA361f CYA785r | 390 |  |
|  |  | Isolate |  | Cyanobiont (Cyanobacterium) | ptLSU C-D-rev  SSU-4-forw | 2.300 | (71) |
|  |  | Lichen Thallus |  | Chlorobiont ( Green Algae) | Al1500af  LR3 | 1.400 | Standard |
| Cyanolichen | *Peltula* sp. | Lichen Thallus | Stone, South Africa | Mycobiont (Fungus) | ITS1  LR3 | 1.300 | Standard |
|  |  |  |  | Cyanobiont  (Cyanobacterium) | CYA361f CYA785r | 390 |  |
| Chlorolichen | *Buellia* sp. (LC1-1; LC1-2; LC1-3) | Biocrust | Atacama Desert, Chile | Mycobiont (Fungus) | ITS1  LR3 | 1.300 | Standard |
|  |  |  |  | Chlorobiont (Green Algae) | Al1500af  LR3 | 1.400 | Standard |
| Cyanobacterium | *Microcoleus steenstrupii* KC2-4 | Biocrust | Atacama Desert, Chile | Cyanobacterium | CYA361f CYA785r | 390 | Standard |
| Cyanobacterium | *Nostoc* sp. D8C1-6 | Biocrust | Spitsbergen, Arctic | Cyanobacterium | CYA361f CYA785r | 390 | Standard |
| Chlorolichen | *Psoroma hypnorum* F4-7; M17-8 | Biocrust | Spitsbergen, Arctic | Mycobiont (Fungus) | ITS1  LR3 | 1.300 | Standard |
|  |  |  |  | Chlorobiont (Green Algae) | Al1500af  LR3 | 1.400 | Standard |
| Chlorolichen | *Cladonia* sp. G4-9 | Biocrust | Spitsbergen, Arctic | Mycobiont (Fungus) | ITS1  LR3 | 1.300 | Standard |
| Chlorolichen | *Ochrolechia tartarea* D6-10 | Biocrust | Spitsbergen, Arctic | Mycobiont (Fungus) | ITS1  LR3 | 1.300 | Standard |
| Chlorolichen | *Cetrariella delisei* sp. H4-11 | Biocrust | Spitsbergen, Arctic | Mycobiont (Fungus) | ITS1  LR3 | 1.300 | Standard |
| Chlorolichen | *Cetraria islandica* FI5-14 | Biocrust | Spitsbergen, Arctic | Mycobiont (Fungus) | ITS4  ITS5 | 800 | Standard |
| Bryophyte | *Dicranum spadiceum* | Biocrust | Spitsbergen, Arctic | Bryophyte | trnVR  trnMF | 750 | Standard |
| Bryophyte | *Hypnum bambergeri* | Biocrust | Spitsbergen, Arctic | Bryophyte | trnVR  trnMF | 750 | Standard |

**Table S2**

|  | **Target** | **Primer** | **Sequence** | **Reference** | **Cycle** |
| --- | --- | --- | --- | --- | --- |
| **Cyanobacteria** | partial 16S-23S rRNA; taxonomy | Boyer1 | CTC TGT GTG CCT AGG TAT CC | (78) | Standard |
|  |  | Boyer2 | GGG GGA TTT TCC GCA ATG GG |  |  |
|  | phaC/E; PHB production | phaC(3.1)-F | GGG ATG TCT ATT TGA TTG AYT GG | (79) | (79) |
|  |  | phaC(3.1)-R | GGT CGG GAC TAT CAA AAA TCC A |  |  |
|  | sxtB; Saxitoxin | SXTB-F | TTT GTA GGR CAG GCA CTT | (80) | Standard |
|  |  | SXTB-R | ATC ATC GGT ATC ATC GGT A |  |  |
|  | mcyE; Mycrocystin | mcyE-F2 | GAA ATT TGT GTA GAA GGT GC | (81) | Standard |
|  |  | mcyE-R4 | AAT TCT AAA GCC CAA AGA CG |  |  |
| **Fungi** | RPB1 | RPB1-VHAFasc | ADT GYC CYG GYC ATT TYG GT | (82) | (83) |
|  |  | RPB1-VH6R | ATG ACC CAT CAT RGA YTC CTT RTG |  |  |
|  | RPB2 | fRPB2-5F | GAY GAY MGW GAT CAY TTY GG | (84) | (84) |
|  |  | fRPB2-7CR | CCC ATR GCT TGY TTR CCC AT |  |  |
| **Green Algae** | 18S rDNA; *Trentepohlia*-specific | Tre18S_N1_for | CCC GAC CTT CGG TGA ATC | (85) | Standard |
|  |  | CHtrente1.rev | CCA CCT CCG ATC CCT AGT |  |  |
|  |  | Tre18S_N5_for | GGA TGA CAC GAT AGG ACT TCG |  |  |
|  |  | CHtrente2.rev | ACA AAG CTC TAG CCC CAT CA |  |  |
|  | ITS; *Trentepohlia*-specific | ITS4_Tre | TCC TCC GCT TAT TGA TAT GC | (85) | Standard |
|  |  | CHtrente2.for | TGA TGG GGC TAG AGC TTT GT |  |  |
|  | rbcL; *Trentepohlia*-specific | TrerbcL_mos_for | GAA GCW ATT CCR GGA GAA G | (85) | Standard |
|  |  | TrerbcL_mos_rev | CAT CCA TTC TTG AGW AAA GAA TAC |  |  |

**References**

78. Boyer SL, Flechtner VR, Johansen JR. 2001. Is the 16S–23S rRNA internal transcribed spacer region a good tool for use in molecular systematics and population genetics? A case study in cyanobacteria. *Molecular biology and evolution*, 18(6), 1057-1069.

79. Lane CE, Benton MG. 2015. Detection of the enzymatically-active polyhydroxyalkanoate synthase subunit gene, phaC, in cyanobacteria via colony PCR. *Molecular and cellular probes*, 29(6), 454-460.

80. Hoff-Risseti C, Dörr FA, Schaker PDC, Pinto E, Werner VR, Fiore MF. 2013. Cylindrospermopsin and Saxitoxin Synthetase Genes in *Cylindrospermopsis raciborskii* Strains from Brazilian Freshwater. *PLoS ONE*, 8, e74238.

81. Rantala A, Fewer DP, Hisbergues M, Rouhiainen L, Vaitomaa J, Börner T, Sivonen K. 2004. Phylogenetic evidence for the early evolution of microcystin synthesis. *Proceedings of the National Academy of Sciences*, 101(2), 568-573.

82. Hofstetter V, Miadlikowska J, Kauff F, Lutzoni F. 2007. Phylogenetic comparison of protein-coding versus ribosomal RNA-coding sequence data: a case study of the Lecanoromycetes (Ascomycota). *Molecular phylogenetics and evolution*, 44(1), 412-426.

83. Vilgalys, R., and Hester, M. 1990. Rapid genetic identification and mapping of enzymatically amplified ribosomal DNA from several Cryptococcus species. *Journal of bacteriology*, 172(8), 4238-4246.

84. Liu YJ, Whelen S, Hall BD. 1999. Phylogenetic relationships among ascomycetes: evidence from an RNA polymerse II subunit. *Molecular biology and evolution*, 16(12), 1799-1808.

85. Hametner C, Stocker‐Wörgötter E, Rindi F, Grube M. 2014. Phylogenetic position and morphology of lichenized Trentepohliales (Ulvophyceae, Chlorophyta) from selected species of Graphidaceae. *Phycological Research*, 62(3), 170-186.
